# Understanding the role of disease knowledge and risk perception in shaping preventive behavior for selected vector-borne diseases in Guyana

**DOI:** 10.1371/journal.pntd.0008149

**Published:** 2020-04-06

**Authors:** Céline Aerts, Mélanie Revilla, Laetitia Duval, Krijn Paaijmans, Javin Chandrabose, Horace Cox, Elisa Sicuri

**Affiliations:** 1 ISGlobal, Hospital Clínic, Universitat de Barcelona, Barcelona, Spain; 2 Research and Expertise Centre for Survey Methodology, Universitat Pompeu Fabra, Barcelona, Spain; 3 Centre d’Economie de la Sorbonne, University Paris 1 Panthéon-Sorbonne; 4 Center for Evolution and Medicine, School of Life Sciences, Arizona State University, Tempe, Arizona, United States of America; 5 The Biodesign Center for Immunotherapy, Vaccines and Virotherapy, Arizona State University, Tempe, Arizona, United States of America; 6 Vector Control Services, Ministry of Public Health, Georgetown, Guyana; 7 Health Economics Group, Department of Infectious Disease Epidemiology, School of Public Health, Imperial College London, London, United Kingdom; Centers for Disease Control and Prevention, UNITED STATES

## Abstract

**Background:**

Individual behavior, particularly choices about prevention, plays a key role in infection transmission of vector-borne diseases (VBDs). Since the actual risk of infection is often uncertain, individual behavior is influenced by the perceived risk. A low risk perception is likely to diminish the use of preventive measures (behavior). If risk perception is a good indicator of the actual risk, then it has important implications in a context of disease elimination. However, more research is needed to improve our understanding of the role of human behavior in disease transmission. The objective of this study is to explore whether preventive behavior is responsive to risk perception, taking into account the links with disease knowledge and controlling for individuals’ socioeconomic and demographic characteristics. More specifically, the study focuses on malaria, dengue fever, Zika and cutaneous leishmaniasis (CL), using primary data collected in Guyana–a key country for the control and/or elimination of VBDs, given its geographic location.

**Methods and findings:**

The data were collected between August and December 2017 in four regions of the country. Questions on disease knowledge, risk perception and self-reported use of preventive measures were asked to each participant for the four diseases. A structural equation model was estimated. It focused on data collected from private households only in order to control for individuals’ socioeconomic and demographic characteristics, which led to a sample size of 497 participants. The findings showed evidence of a bidirectional association between risk perception and behavior. A one-unit increase in risk perception translated into a 0.53 unit increase in self-reported preventive behavior for all diseases, while a one-unit increase in self-reported preventive behavior (i.e. the use of an additional measure) led to a 0.46 unit decrease in risk perception for all diseases (except CL). This study also showed that higher education significantly improves knowledge and that better knowledge increases the take up of preventive measures for malaria and dengue, without affecting risk perception.

**Conclusions:**

In trying to reach elimination, it appears crucial to promote awareness of the risks and facilitate access to preventive measures, so that lower risk perception does not translate into lower preventive behavior.

## Author summary

In the context of Guyana, people’s self-reported behavior (i.e. use of vector control tools) is based on their risk perception and on their knowledge of the disease if the risk perception is high enough (i.e. for malaria and dengue fever). Measures donated by the government, such as bed nets, are widely reported to be used and their use is less likely to be contingent on the perceived risk. In other words, because those measures are donated, they are more likely to be used regardless of the risk perception. The type of region in which individuals live also plays a key role on the adoption of vector control measures: although people living in the hinterland tend to have greater knowledge about the disease and an accurate risk perception, they use fewer preventive measures than people living in the coastal regions–thus pointing to the importance of promoting access to preventive measures in the hinterland. Therefore, in trying to reach the elimination of vector-borne diseases, it is essential for the government to promote awareness of the risks and facilitate (i.e. donate) access to preventive measure to avoid a reduced usage of vector control measures arising from a lower risk perception.

## Introduction

According to the World Health Organization (WHO), vector-borne diseases (VBDs) represent 17% of all infectious diseases and cause more than 700,000 deaths annually, with 80% of the world’s population at risk [1,2]. VBDs are caused by pathogens transmitted through vectors, most of them being bloodsucking insects such as mosquitoes or sandflies. Since 2014, major outbreaks of VBDs such as malaria, dengue fever, Zika and chikungunya have spread to previously unaffected areas of Latin America, overwhelming the health system of many countries [1,3–6]. Individual behavior–particularly choices about prevention–plays a role in infection transmission, and is thus a topic of interest in both the public health and social science (e.g. economic) disciplines. Yet, more research is needed to improve our understanding of the role of human behavior in disease transmission so that policy-making translates into decreased morbidity and saved lives [7–9].

In the public health discipline, behavioral practice is often studied together with knowledge and risk perception through ‘knowledge, attitude, and practice’ (KAP) surveys. Although KAP studies are informative, they are generally descriptive and do not dig in the complex links between knowledge, risk perception and behavioral practices. No KAP studies have been previously carried out in Guyana on VBDs but several have been carried out in other contexts. Keeping in mind that these contexts are different from Guyana, the results of such studies–among others (i.e. mixed method and qualitative studies)–are inconclusive regarding the association between knowledge and behavior: some find a positive association [10–15], whereas other find a negative [16] or no association [17–22]. This diversity in the findings suggests that the results are context specific and cannot be generalized across different areas/regions and diseases. The most similar context in which a KAP study has been conducted was in French Guyana, and it shows that an increased understanding of transmission led to better dengue prevention practices [11]. Moreover, while KAP studies do not shed light on the association between risk perception and behavior, other quantitative studies generally report a lack of association between the two. For instance, a recent study conducted by Chan et al. shows that (using the Granger causality test) there is no association between risk perception and protective behavior against Zika in the United States (US) [23]. Similarly, through a confirmatory factor analysis, Castro et al. find no association between greater risk perception and dengue related practices in Cuba [12].

In the economic discipline, behavior is usually modelled as the demand for prevention, which is assumed responsive to risk perception. More precisely, when purchasing preventive measures, individuals estimate the costs of prevention against the benefits of avoiding the infection in the future. As the actual risk, expressed in terms of prevalence or incidence, is often uncertain–if not completely unknown by the population at risk–prevention decisions are affected by the individual’s risk perception and preferences [8]. Risk-averse individuals will face a ‘risk-elastic demand for prevention’: a percentage increase in the risk will lead to a greater percentage increase in self-protective behavior. The demand is also more likely to be ‘risk-elastic’ when vaccines are inexistent; and yet more if treatment options are lacking, inadequate or unaffordable [24]. Quantifying the elasticity of the demand to the risk perception is essential for effective prevention programs because it will predict the effect of changes in the risk perception on individual choices. Elastic demand to risk perception makes it harder to eradicate a disease: as the transmission of the infection decreases, risk perception should decrease and even more so should the demand for prevention [8]. Risk-elastic demand is supported by theoretical economic models but in reality, few empirical studies have looked into it. A majority focus on HIV [8,25–28] but few on VBDs. For malaria, a reference study is the one by Picone et al., which looked at the elasticity of bed nets usage for malaria across nine countries in sub-Saharan Africa and finds a coefficient that is positive but lower than one, suggesting an inelastic relationship [29]. Two main factors can explain the scarcity of quantitative research on the topic: the (i) challenges in measuring behavior and (ii) reverse relationship between behavior and risk perception [8]. Behavior is often self-reported as it is difficult to observe and measure [30]; self-reported behavior tends to overestimate actual behavior due to–among others–social desirability bias [31]. The second issue affecting quantitative/statistical modelling is the reverse relationship between behavior and risk perception: more precisely, it is difficult to estimate the impact of risk perception on preventive behavior if the same behavior in the past has contributed to today’s risk perception [8]. Unless using appropriate research designs or statistical methods, the response of behavior to risk is likely to be biased upward [32]. A common way to deal with endogeneity is using an instrumental variable approach but finding a robust instrument may be challenging. Another way suggested to overcome this statistical challenge is using a structural equation model (SEM), which is able to control for over-reporting of preventive behavior and isolate the impact of risk perception on behavior and vice versa.

The objective of this study is to assess whether preventive behavior is responsive to risk perception and whether it differs across diseases, taking into account the role of disease knowledge on behavior and risk perception, and controlling for individuals’ socioeconomic and demographic characteristics. The innovative character of this study lies in its focus on four diseases (malaria, dengue, Zika and cutaneous leishmaniasis (CL)) and in its reliance on primary data collected in Guyana, where practically no research has ever been conducted on the topic. Moreover, these four VBDs are responsible for a significant morbidity and mortality burden worldwide [1,33–35]. The measure of the burden for these diseases (except for malaria) is limited in Guyana due to a deficient surveillance system. Nonetheless, according to the Ministry of Public Health (MoPH), they are responsible for a substantial morbidity burden. Despite the country’s small population, the number of malaria cases in Guyana accounts for 3% of the total estimated cases in America, with incidence levels in specific areas of the hinterland (i.e. mining areas) that are above many sub-Saharan African countries [36].

## Material and methods

### Ethics statement

The study protocol with the reference number 265 was reviewed and approved by the Institutional Review Board (IRB) of the Ministry of Public Health of Guyana. All adult subjects provided written informed consent prior to participating to the study.

### Study setting and population

Guyana lies between Suriname, Brazil and Venezuela, spanning over 216,000 square kilometers with a total population of approximately 780,000 inhabitants [37]. It is divided into ten administrative regions, which are either categorized as so-called hinterland or coastal based on their geographical location, demographic characteristics, soil type, economic activities, and natural resources, among others. Coastal regions are more densely populated and include the capital city Georgetown (region 4), where nearly half of the country’s population live [38]. While some infectious diseases are more prevalent in the coastal regions and others in the hinterland, a surveillance system reporting the exact distribution and number of cases across the country is only available for malaria. The number of malaria cases have increased for the two most recent figures, with 11,108 reported cases in 2016 and 13,936 cases in 2017. Figures available for dengue report 230 laboratory-confirmed cases in 2019, 286 cases in 2018, while up to 863 cases in 2014 [39,40]. As for Zika, 52 cases have been reported in 2015, 339 in 2016 while none in 2017 [41]. One figure is available for CL in 2017, which reports 21 confirmed cases per 100,000 inhabitants; its incidence rate varies greatly across the country and is classified as ‘intense’ in the hinterland [42]. The data collected by the MoPH suggest that Zika and dengue are more prevalent in the coastal regions whereas CL and malaria are more prevalent in the hinterland, where mining areas are the hot spot of infection. Overall, infectious and parasitic diseases are estimated to be responsible for 11% of the deaths in the country [40]. Importantly, Guyana is a strategic country given its geographic location for promoting the control and elimination of VBDs in the Northern coast of South America and the Caribbean. A regional cooperation between the Guianas (Guyana, Suriname and French Guyana) and Brazil has often been reported to be necessary [43–45]. Moreover, Guyana shares the border with Venezuela, which is facing a difficult political situation and experiencing an overwhelming resurgence of VBDs transmission [6].

### Data collection and analysis

In Guyana, not all regions are endemic or equally endemic. Therefore, the data were collected in four regions of the country, two in the hinterland (regions 1 and 8) and two along the coast (regions 4 and 6) to capture endemic and non-endemic areas depending on the disease. In the coastal regions (4 and 6, populated and urban areas), 15 villages per region were randomly selected (among preselected villages by the MoPH for their reachability by foot or public transports) based on ‘selection proportional to size’. This method randomly selected villages based on (i) the chosen number of villages per region and on (ii) the number of inhabitants per village. Within those 15 villages, the number of questionnaires administered was also proportional to size: the most populated village had the highest number of questionnaires assigned and vice versa (cf. S2 File). In the hinterland regions (1 and 8), given the very low number of inhabitants, ‘selection proportional to size’ was not applied. Instead, villages with the highest population density (that include the main health facilities, our starting point for data collection) were selected to participate and questionnaires were administered until the target size was reached. This being said, in all regions and within all selected villages, data collectors selected houses starting from the health facility and moving forward while applying the ‘spinning bottle’ rule [46].

**Fig 1:**
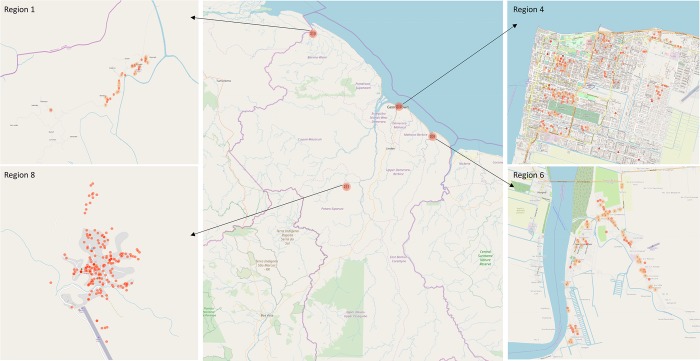
Map of data collection. Each dot contains the number of individuals interviewed in that specific area. Note that while region 1 may appear as coastal, it is classified as hinterland by the government given its economic activity and topography. Source: the map was created from the data we collected using the KoBoToolbox.

Given the available resources, the targeted sample size was set to above 800 in total: 210 questionnaires per region. Accordingly, between 209 and 215 participants (over 18 years old) were interviewed in each region (Fig 1). Before conducting the interviews, a training of the fieldworkers (data collectors) was organized, followed by a piloting of the questionnaire in each region. The questionnaire was then refined based on fieldworkers’ feedback. Face-to-face interviews were performed using tablets and conducted from August 2017 to December 2017. These were conducted in private house (59%), workplaces (30%), schools (5%), restaurants (4%), and in hospitals/health centers (2%). In private houses, as opposed to the other places, a set of indicators such as assets and livestock ownership, education and occupation was gathered (cf. S1 File). We focused our analysis on data collected from private houses only (59%) to be able to control for the individuals’ socioeconomic and demographic characteristics (N = 497). In order to capture disease knowledge, risk perception and behavior for the four diseases separately, the following questions were asked to each of the participants:

- “Do you know disease X?”
  If the respondent answered ‘yes’, he/she was asked:
  “Can you briefly describe what you know about disease X?”
- “How much do you think you and the people in this place are at risk of disease X on a scale from 0 to 10 (0 –no risk; 10 –very high risk)?”
- “What do you do to avoid disease X?”

Keywords for describing the diseases and preventive tools were selected based on discussions with the MoPH. As participants were describing the diseases or reporting their behavioral practices, a box was ticked for each keyword mentioned. The possible answers for behavioral practices were mainly related to the use of preventive tools (cf. S1 File).

The data were uploaded in an online (secured) reporting platform that guaranteed anonymity of the data. The analysis was conducted in Stata software (StataCorpLP, http://www.stata.com) to obtain the correlation matrices that were then inputted in LISREL (http://www.ssicentral.com/lisrel/) to estimate the SEM [47] (cf. S1 Text).

### Descriptive data

Descriptive statistics of the respondents across regions are presented in Table 1. The socioeconomic status (SES) consists in a wealth index that was obtained by applying multiple correspondence analysis (MCA) to asset and livestock ownerships. A wealth index per region was initially computed since asset and livestock ownerships may have different meanings to wealth across regions. For instance, livestock ownership may be a sign of richness in the hinterland (i.e. regions 1 and 8) while the opposite in the capital city (i.e. region 4). S1 Fig shows the dimension 1 and 2 of the MCA per region. Dimension 1 is interpreted as wealth: modalities with negative coordinates can be seen as indicators of ‘richness’ whereas positive coordinates are indicators of ‘poorness’. For instance, for region 4, not having electricity, a color-television and/or a refrigerator is clearly a sign of low economic status (cf. S1 Fig). The four wealth indices were then pooled into one index. If looking across regions, there is a higher proportion of people belonging to the first quintile (i.e. the poorest) of the wealth index in regions 1 and 8, the hinterland. As expected, regions that recorded the highest proportion of its population in the first quintile also experienced the highest rate of ‘no formal education’. Lastly, a significant majority of the participants consisted of female for interviews conducted in private houses.

**Table 1:** Descriptive statistics of the respondents interviewed in private houses.

	Hinterland		Coastal regions	
	Region 1 N (freq.)	Region 8 N (freq.)	Region 4 N (freq.)	Region 6 N (freq.)
**Wealth index**				
1^st^ quintile (poorest)	36 (24.49%)	28 (24.35%)	16 (12.21%)	20 (19.23%)
2^nd^ quintile	37 (25.17%)	16 (13.91%)	29 (22.14%)	17 (16.35%)
3^rd^ quintile	17 (11.56%)	19 (16.52%)	36 (27.48%)	30 (28.84%)
4^th^ quintile	28 (19.05%)	22 (19.13%)	26 (19.85%)	22 (21.15%)
5^th^ quintile (richest)	29 (19.73%)	30 (26.09%)	24 (18.32%)	15 (14.42%)
**Education**				
No formal education	8 (5.44%)	3 (2.61%)	0	0
Primary	47 (31.97%)	45 (39.13%)	28 (21.37%)	25 (24.04%)
Secondary	82 (55.78%)	64 (55.65%)	99 (75.57%)	67 (64.42%)
University	10 (6.80%)	3 (2.61%)	4 (3.05%)	12 (11.54%)
**Sex**				
Female	99 (67.35%)	93 (80.87%)	109 (83.21%)	76 (73.08%)
Male	48 (32.65%)	22 (19.13%)	22 (16.79%)	28 (26.92%)
**Sample size (N)**	147	115	131	104

Legend: freq = frequency.

### The model

**Fig 2:**
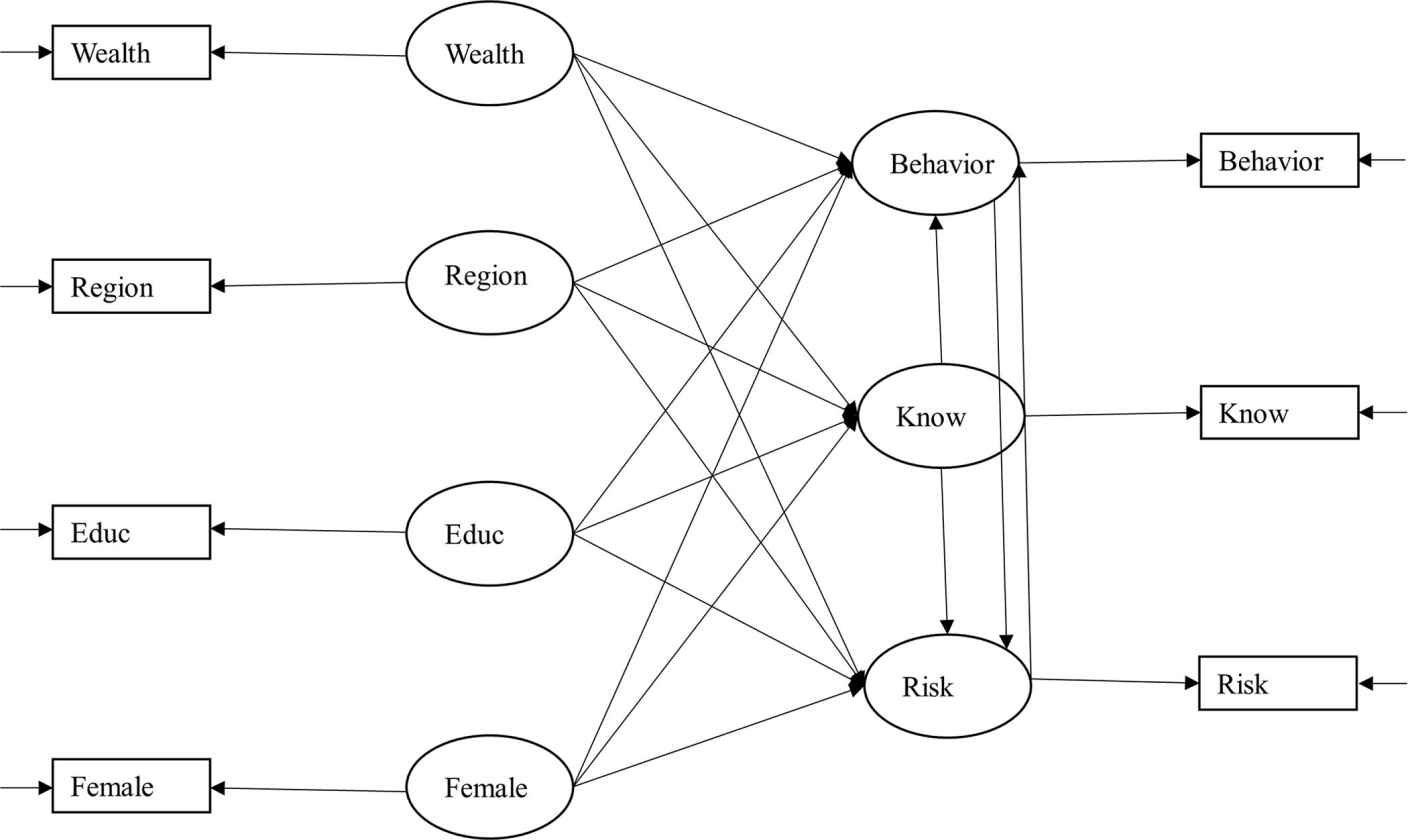
Path diagram of the system of simultaneous equations. Circled variables are the latent ones and boxed variables are the observed ones. The arrow from the circled variables to the boxed variables indicates the quality coefficient.

SEM offers several advantages: it can (i) deal with omitted variable bias [48]; (ii) account for measurement error by using latent variables as indicators of observed variables [49]; (iii) solve for reverse relationship if the model is empirically identified; and (iv) compare models in terms of their best fit as well as perform multiple group analysis [50,51] (cf. S3 Text) Our SEM model is presented in Fig 2 and is built assuming a linear structure of relationships using the maximum likelihood estimator (MLE) for participants interviewed in private houses. It was developed based on findings from the literature, mainly from KAP studies. The variable knowledge is a categorical variable, which is equal to 0 if the person does not know the disease; 1 if the person cited one keyword; 2 if the person cited two keywords; etc. People who reported absolutely no knowledge of the disease were removed from the analysis since we cannot obtain an unbiased estimate of their risk perception and behavior. We nonetheless included in S3 File a probit estimation to assess the determinants of knowledge across the four diseases. The variable behavior is also categorical, measured as the reported number of vector control strategies used by the respondent. In this analysis, behavior embeds two types of tools: ‘personal protection tools’ (e.g. mosquito coils and repellent) and ‘vector control tools’ (e.g. fogging and IRS). It nevertheless does not look at ‘vector reduction behavior’ such as water holding container management. In coding behavior, we distinguished between ‘passive’ and ‘active’ behavior, where the former captures usage only and the latter captures demand. More precisely, ‘passive’ behavior refers to using measures that were provided free of charge by the MoPH while ‘active’ behavior refers to using measures that were purchased. Accordingly, ‘passive’ behavior includes bed nets (treated with 55mg/m^2^ deltamethrin), indoor residual spraying (IRS) and fogging (all provided by the MoPH in Guyana) and ‘active’ behavior includes tools such as mosquito coils, skin repellent, screened windows–among others. Hence, the variable behavior is equal to 0 if the person reports to use nothing (although the person knows about the disease); 1 if the person ‘uses’ only one or all of the measures provided by government (a bed net and/or IRS and/or fogging); 2 if the person uses one other measure than the ones provided by the government; 3 if the person uses two other measures than the ones provided by the government; etc. Usage of measures such as fogging or IRS should be interpreted as acceptation of invasive but necessary interventions inside and around the house. Nevertheless, in order to test the robustness of our definition of ‘positive’ behavior, we ran the model with an alternative definition in which bed net use is considered as active and not passive, even if not purchased but received free of charge from the government. In that case, the dichotomy between active and passive behavior is not based on the measures being purchased/donated but on them requiring an active usage versus a passive one. Accordingly, the variable behavior is equal to 0 if the person reports to use nothing; 1 if the person ‘uses’ IRS and/or fogging; 2 if the person uses another measure than IRS and/or fogging; etc. The variable risk is a measure of self-reported risk perception and ranges from 0 to 10, with 0 meaning ‘no risk’ and 10 meaning ‘very high risk’ as indicated by the question. As for the exogenous variables, the variable wealth consists of a wealth index. The variable educ stands for education and ranges from no formal education to university degree–‘no formal education’, ‘primary’, ‘secondary’, and ‘university’. The variable region is a dummy variable taking the value of 1 if the person lives in the hinterland (regions 1 or 8), and 0 if the person lives in a coastal region (4 or 6). The variable female is equal to 1 if the respondent is a female and 0 otherwise.

Where the algebraic representation of the SEM is as follow:

$$\text{Behavior}=\beta_{1}\text{knowledge}+\beta_{2}\text{risk}+\beta_{3}\text{wealth}+\beta_{4}\text{region}+\beta_{5}\text{educ}+\beta_{6}\text{female}+\varepsilon_{\text{B}}$$ (1)

$$\text{Knowledge}=\gamma_{1}\text{wealth}+\gamma_{2}\text{region}+\gamma_{3}\text{educ}+\gamma_{4}\text{female}+\varepsilon_{\text{K}}$$ (2)

$$\text{Risk}=\delta_{1}\text{knowledge}+\delta_{2}\text{behavior}+\delta_{3}\text{wealth}+\delta_{4}\text{region}+\delta_{5}\text{educ}+\delta_{6}\text{female}+\varepsilon_{\text{R}}$$ (3)

For which E(ε_B_, ε_K_, ε_R_) = 0.

Eq (1): As mentioned in the introduction, the findings on the association between knowledge and behavior cannot be generalized across diseases and contexts, while for risk perception, economic models believe it to increase behavior but few empirical studies have proved so. As for wealth, it is expected to have a positive effect on behavior through greater purchasing power [52]. Higher level of education also tends to increase the demand for preventive tools [16,52,53] and women have a tendency to adopt a greater range of vector control practices than men [16,53].

Eq (2): Disease knowledge is believed to be determined primarily by education and higher wealth from increased access to information through multiple channels such as television, radio and social media [10,12,17,52,54]. Sex also tends to play a role on knowledge: a study demonstrated that women had 63% higher odds of being able to correctly cite at least three dengue symptoms [17].

Eq (3): Higher knowledge is likely to be associated with greater risk perception [18] [23] although some studies found otherwise [11] [13]. Safer behavior is supposed to decrease risk perception from a feeling of control over the infection [7]. People with a higher economic status are more likely to have a more accurate perception of the risk, potentially through higher education and knowledge [55]. As for the sociodemographic variables educ and *female*, they tend to play a role in risk perception but findings are mixed [10,17,55].

Lastly–as for the other socioeconomic and demographic variables–the variable region is included in each equation. Preventive behavior, knowledge and risk perception are all likely to vary depending on the geographic proximity to the diseases’ vectors but also on other characteristics that are specific to the region (e.g. education system, health system, transportation services, etc.) [22] [53].

### Measurement error and testing of the model

Measurement errors in data collected through surveys can be significant, implying a significant margin between the variable that one wishes to measure and the one that is truly measured [56,57]. As a result, measurement was considered for the self-reported variables: behavior, knowledge and risk perception using the Survey Quality Predictor program (http://sqp.upf.edu/) [58]. To test the model’s fit, a postestimation tool for the SEM–Jrule–was used in addition to the usual chi-square test to identify potential local misspecifications [59–61] (cf. S4 Text).

## Results

### Descriptive results

#### Disease knowledge

As seen in Table 2, almost 80% of the population did not know about CL, whereas this figure was about 54% for Zika, 33% for dengue and 12% for malaria. The determinants of knowledge–obtained through a probit model–varied across diseases (cf. S3 File). Yet, a significant determinant of knowledge across diseases was the variable region: people living in the hinterland (regions 1 and 8) had a higher probability of knowledge for malaria and CL. Furthermore, people living in region 1 had a higher probability of knowledge not only for malaria and CL but for Zika and dengue as well. In addition to this, a higher education level increased the probability of knowledge for Zika and malaria but did not significantly affect CL or dengue; being a male increased the likelihood of knowing about malaria and dengue; and pertaining to a higher quintile of the wealth index increased the probability of knowing about Zika and CL but not about dengue and malaria.

**Table 2:** Knowledge level per disease.

*Do you know about the disease? If yes, can you please describe what you know about the disease*?				
	Malaria	Dengue fever	Zika	Cutaneous leishmaniasis
**Does not know**	**11.87%**	**32.60%**	**53.52%**	**77.46%**
At least 1 keyword cited	88.13%	67.40%	46.48%	22.54%
At least 2 keywords cited	70.82%	32.19%	23.54%	4.43%
At least 3 keywords cited	50.50%	3.22%	10.66%	
At least 4 keywords cited	29.78%		3.82%	
At least 5 keywords cited	16.10%			
At least 6 keywords cited	11.07%			

Legend: for each disease, the number of keywords cited was summed up.

Afterwards, for the individuals who reported a minimum knowledge of the diseases, their level of knowledge–based on the number of keywords cited regarding the diseases’ causes and symptoms–was measured. From Table 2, one can see that up to 6 keywords could be cited for malaria while only two for CL. Knowledge of CL (called ‘bush yaws’ by the population) was low, potentially because the disease mainly affects a subsample of the population which are gold miners. For a description of the keywords cited, refer to S1 Table.

### Risk perception

**Fig 3:**
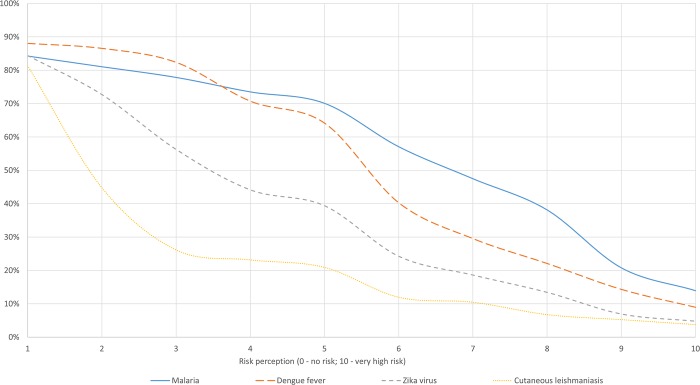
Cumulative frequency of self-reported risk perception across diseases. As this graph shows the cumulative frequency of risk perception, we start by including the people who had a risk perception of at least 1 (on a scale from 0 to 10). The percentage of people who had a risk perception of 0 can be computed for each disease by subtracting to the sample the proportion of people who perceived a risk of at least 1. For instance, for malaria, 100%-84% = 16% of the sample believed the risk to be 0 (although knowing about the disease).

Fig 3 shows the cumulative frequency of self-reported risk perception per disease. Depending on the disease, between 12% and 18% of the respondents believed the risk of infection to be zero while between 3% and 14% believed the risk to be 10. Risk perception and knowledge seemed to follow the same path: the median risk perception is the highest for malaria, followed by dengue, Zika and CL.

### Disease practices

Self-reported usage of vector control tools for each disease is tabulated in Table 3. When people know about the disease, they tend to protect themselves–except for CL,for which almost 55% of the population reported to do nothing to protect against the vector. Measures provided by the government were highly used among the population. About 94% for malaria, 90% for dengue and 81% for Zika of the respondents reported to use at least one measure offered by the government (i.e. a bed net and/or IRS and/or fogging). For the individuals who only used the measures donated by the government, bed net seemed the most common one. For the remaining individuals, overall, they used a maximum of three additional measures–or two in the case of CL–than the ones provided by the government. Among these, skin repellent and mosquito coils were the most commonly purchased ones (cf. S2 Table).

**Table 3:** Self-reported vector control practices.

*What do you do to avoid disease x*?					
First definition of behavior (i.e. bed net use is passive)					
		Malaria	Dengue fever	Zika	Cutaneous leishmaniasis
	**Nothing**	3.88%	7.16%	3.46%	54.48%
Passive	Bed net and/or IRS and/or fogging only	45.43%	57.01%	39.39%	29.85%
Active	Use 1 other measure than a bed net/IRS/fogging	34.02%	29.85%	47.62%	12.69%%
	Use 2 other measures than a bed net/IRS/fogging	14.38%	5.67%	8.23%	2.99%
	Use 3 other measures than a bed net/IRS/fogging	2.28%	0.3%	1.30%	
**Second definition of behavior (i.e. bed net use is active)**					
	**Nothing**	3.88%	7.16%	3.46%	54.48%
Passive	IRS and/or fogging only	4.34%	19.19%	9.96%	18.66%
Active	Use 1 other measure than a IRS/fogging	44.29%	42.69%	49.35%	14.18%
	Use 2 other measures than a IRS/fogging	31.28%	25.37%	30.74%	9.70%
	Use 3 other measures than IRS/fogging	14.38%	5.67%	5.63%	2.99%
	Use 4 other measures than IRS/fogging	1.83%		0.87%	

Legend: IRS = Indoor residual spraying.

In the first definition of behavior, passive behavior includes all measures that are donated by the government (IRS, fogging and bed nets). In the second definition of behavior, passive behavior only includes IRS and fogging but not bed nets since it can be seen as requiring an ‘active’ usage.

### SEM results

Standardized estimates of the structural model are reported in Table 4 and the final LISREL input is provided in S2 Text. A quality coefficient of 0.728, 0.744 and 0.752 was estimated for behavior, knowledge and risk respectively, implying a measurement error of approximately 25%. The process that led to those measurement errors can be traced in the SQP database under the study name of ‘repuls’. Following Jrule postestimation results, the effect of region on risk perception was let free to vary across the four diseases. Other parameters were let free to vary but for some specific diseases only, such as knowledge on behavior for CL and Zika; knowledge on risk perception for CL; etc. This led to a model with a chi-square of 31.99, 24 degrees of freedom and an associated p-value of 0.12714, which combined with a Root Mean Square Error of Approximation (RMSEA) of 0.034, suggested that the model fits well the data.

**Table 4:** Results of the structural model.

		Malaria	Dengue fever	Cutaneous leishmaniasis	Zika
Dependent variable	Explanatory variables	St. Coeff (Std. Error)	St. Coeff (Std. Error)	St. Coeff (Std. Error)	St. Coeff (Std. Error)
**Eq 1**					
Behavior	Knowledge	0.841*** (0.106)	=	0.747 (0.554)	0.203 (0.189)
	Risk	0.530** (0.232)	=	=	=
	Wealth	0.0117** (0.057)	-0.121 (0.080)	=	=
	Region	-0.841*** (0.169)	=	-0.172 (0.338)	-0.119 (0.147)
	Educ	0.006 (0.052)	=	=	=
	Female	0.010 (0.045)	=	=	=
**Eq 2**					
Knowledge	Wealth	0.001 (0.045)	=	-0.177** (0.085)	0.177** (0.077)
	Region	0.639*** (0.059)	0.380*** (0.066)	-0.589*** (0.095)	0.568*** (0.077)
	Educ	0.256*** (0.040)	=	0.051 (0.066)	=
	Female	0.019 (0.033)	=	=	=
**Eq 3**					
Risk	Knowledge	0.28 (0.185)	=	1.503 (1.089)	=
	Behavior	-0.463** (0.212)	=	-0.010 (0.876)	=
	Wealth	0.123* (0.064)	-0.099 (0.085)	=	-0.007 (0.104)
	Region	0.695*** (0.106)	0.211 (0.153)	=	-0.194 (0.146)
	Educ	0.056 (0.052)	0.056 (0.052)	=	-0.015 (0.106)
	Female	0.009 (0.040)	=	=	=
N		438	335	134	231

Chi-Squared (df) = 31.99 (24); p-value = 0.12714

RMSEA = 0.034

Legend: ‘=’ implies that coefficients are equal to the ones estimated for the malaria model (model 1);

**significant at 5% significance level;

*** significant at 1% significance level; St. Coeff = standardized coefficient; Std. Error = Standard error; N = sample size; df = degrees of freedom; RMSEA = Root Mean Square Error of Approximation. Region is a dummy variable equal to 1 if the respondent lives in the hinterland and 0 otherwise.

The results showed that behavior was significantly responsive to the level of risk perception: a one-unit increase in risk perception (on a scale from 0 to 10) increased the demand for prevention by 0.53 unit for all diseases. Behavior also seemed to be responsive to the level of knowledge but for malaria and dengue only since for CL and Zika, the coefficient lacked statistical significance. That is, a one-unit increase in disease knowledge (i.e. one more keyword cited) increased the demand for prevention by 0.84 unit for malaria and dengue. The association between wealth and behavior was statistically significant but close to zero (or not statistically significant in the case of dengue) indicating that low purchasing power did not act as a considerable barrier to the adoption of additional measures. Overall, people in the hinterland seemed to demand less for prevention than in coastal regions: more precisely, they demanded 0.84 unit less for malaria and dengue and between 0.12 and 0.17 unit less (although not significant) for Zika and CL respectively. As for education and sex, they showed to have no influence on the demand for prevention.

Regarding the degree of disease knowledge, it varied depending on the respondent’s level of education and the region in which he/she lived. More exactly, people living in the hinterland had greater knowledge about malaria, dengue and Zika (i.e. from 0.38 to 0.64 unit increase) but lower knowledge about CL (i.e. a 0.59 unit decrease). While for education, moving to the next level (e.g. from primary to secondary) increased the level of knowledge by 0.26 unit for all diseases, except for CL, for which the coefficient lacked statistical significance.

With respect to the determinants of risk perception, (i) vector control practices (i.e. behavior), (ii) socio-economic status and (iii) the geographic location seemed key. To be more specific: (i) an additional measure used to protect against the disease decreased risk perception by 0.46 unit for malaria, dengue and Zika but did not affect risk perception for CL; (ii) a higher economic status translated into an increased risk perception (by 0.12 unit) for malaria and CL; and (iii) people living in the hinterland had a higher risk perception for malaria and CL (by 0.70 unit), which, based on the MoPH estimations, would imply that the perceived risk reflected the actual risk of infection. Education and sex had no influence on risk perception for any of the four diseases.

A robustness check on the definition of behavior–where bed net usage is no longer considered passive but active–supported our initial finding of a positive association between risk perception an behavior (cf. S4 File). Furthermore, as expected, the coefficient was smaller in this case: a one-unit increase in risk perception increased the demand for prevention by 0.27 unit, as opposed to 0.53 unit before. This is because considering bed nets use as active in a context in which they are donated diminishes the effect of risk perception on behavior. In other words, because bed nets are donated, they more are likely to be used regardless of the risk perception. This also applies to the reverse relationship–the effect of behavior on risk. Using an additional measure–which can now include bed net–than IRS and/or fogging was no longer statistically significantly associated with a decrease in risk perception. If bed nets are used regardless of the perceived risk, their use are less likely to decrease risk perception. Lastly, while overall our results are similar across the two definitions of behavior, according to the Chi2 test, the model fitted better the data when bed nets use was considered as passive and not active (which seems reasonable in a context in which they are donated).

## Discussion

This study is one of the few empirical ones to show evidence of a circular link between risk perception and preventive behavior [8]. Higher risk perception translates into the take up of more preventive measures–the more people fear, the more they protect themselves–which in turn decreases risk perception. Measures subsidized by the government (specifically bed nets) were highly used showing that–as claimed by Dupas’ work–heavy subsidization of health products promotes their usage [62,63]. Furthermore, as shown by our second definition of behavior, measures that are provided free of charge (i.e. bed nets) were more likely to be used regardless of the perceived risk. This study also demonstrated that, in Guyana, better knowledge increases the take up of personal preventive measures for malaria and dengue without affecting risk perception. This can be explained by the following: the more people know about the diseases, the more measures they will use, the more in control they will feel, and the less affected is their risk perception [7]. As for Zika and CL, better knowledge did not increase the take up of preventive measures, which may be explained by a general low risk perception. As seen in Fig 3, the risk perception was much lower for Zika and CL than for malaria and dengue. This would suggest that if the overall risk perception of a disease is low, greater knowledge is not sufficient to trigger a behavioral change. Therefore, behavior is determined by knowledge if risk perception is high enough. Nevertheless, risk and knowledge were not the only factors to affect behavior. The type of region–hinterland or coastal–in which the respondent lives played an important role. Indeed, throughout the analysis the variable region played a key role in explaining (i) behavior, (ii) knowledge and (iii) risk perception. That is, people in the hinterland (i) used fewer vector control measures but (ii) had a higher knowledge levels for all diseases, except of CL. More specifically, among the people who had a minimum knowledge of the diseases, the level of knowledge (i.e. number of keywords cited) was likely to be higher for all diseases (except CL) for those living in the hinterland. And as seen from the probit estimation, in the hinterland, people were more likely to know about malaria and CL. This overall higher knowledge in the hinterland was the result of greater awareness raising, particularly for malaria, carried out by the MoH to respond to the distance between health facilities and where infection is contracted (i.e. hours/days of travelling) and by gold mining companies to keep their workers healthy and productive. Higher knowledge for dengue and Zika in the hinterland suggested the existence of positive spillover effects of malaria on other VBDs. Nonetheless, we see that this does not apply to CL–of which knowledge was lower in the hinterland–but which could be explained by the bias in our sample: CL is mostly prevalent amongst men working in gold mining camps, while our sample mainly included women. Lastly, people in the hinterland (iii) had a higher risk perception for malaria and CL, where those diseases are actually endemic, thereby indicating that the variable region is a good proxy for the actual prevalence and that the risk perception is consistent with the actual risk of infection. Furthermore, the combination of these three findings–people in the hinterland having a higher knowledge, an accurate perception of the risk but demanding fewer vector control measures–suggested that the variable region was not only a proxy for the disease’s prevalence but captured other features that are specific to the region, such as accessibility. Accessibility to preventive measures is indeed more complicated in the hinterland, and may actually represents a bigger obstacle than wealth to the demand for prevention, which showed to have little effect. Similarly, the variables educ and sex showed to have no influence on the model, except for the effect of education on knowledge. This being said, the lack of statistical significance of the variable sex is likely to be due to an over-representation of women (i.e. 76%) in our sample.

This brings us to the limitations of the study. More than 70% of our sample is made of women simply because they were more likely to be found at home. This over-representation of women in our sample is likely to bias the results–for instance, by lowering the disease knowledge and perceived risk for malaria and CL, which population mostly at risk consists of male gold miners. Another limitation is that we do not know the exact prevalence of dengue, Zika and CL in Guyana, which prevents us from making a direct association between risk perception and the actual risk of being infected. However, we can confirm from the data available that the risk perception for malaria is much greater in regions where the incidence rates are higher. Understanding whether risk perception reflects the true risk of infection is an important line of research that merits further investigation. Additionally, our reliance on cross-sectional data–instead of longitudinal data–implies that we are unable to control for time-invariant characteristics–such as risk preferences–that may influence behavior. Lastly, given the debate on the actual capacity of the SEM to identify causation, we took a conservative position and kept our results to associations rather than causal effects. Beyond this debate, we find SEM as a very useful model when complex and/or circular relationships among variables have to be studied (particularly when cross-sectional data are only available). It also has the advantage of being intuitive and easy to replicate.

To conclude, unpacking the direct and mediating effects of positive behavior against VBDs, we could observe that the perceived risk and the level of knowledge (if the perceived risk is high enough) were key, which were jointly influenced by the geographical location, and individually influenced by current behavior practices and education respectively. Last but not least, it appeared that easier access to preventive measures was also essential to the adoption of vector control measures, which can otherwise undermine the behavioral responsiveness to risk. Thus, accounting for the reverse relationship of behavior on risk perception, we can say that, in the context of Guyana, people act according to the risk they perceived and to their knowledge–if the risk perception is high enough for knowledge to trigger a behavioral change. This finding has important implications for health policy-making, as it can help modelling the impact of outbreaks as well as of public health interventions. Although from this analysis we cannot speak about the elasticity of the demand for prevention to risk, we can confirm that, by providing the population with an accurate estimation of the infection’s risk, the population will respond through greater protection against the vector. Moreover, providing information about the causes and symptoms of the diseases is also likely to increase the take up of preventive measures, especially if the perceived risk is high. While these findings are specific to Guyana, we believe that they can be generalized to some neighboring countries/areas: more specifically, to Suriname, French Guyana and Roraima state in Brazil (which borders region 8 of Guyana that is included in the study).

Consequently, in a context of elimination such as for malaria, one key recommendation from this study is effective communication with the population at risk, particularly during the so-called ‘last mile’. In such a context, for the government and population to act hand in hand, it is essential for the former to promote awareness of the risk to the latter to avoid a decrease in preventive behavior arising from a lower risk perception. This is all the more important for diseases that are asymptomatic or that face common symptoms such of fever and headaches but which lack routine surveillance (e.g. dengue or Zika), as reaching their control and/or elimination is likely to be further challenged by an underestimation of the actual risk of infection. Moreover, as seen in this study, the donation of measures by the government will also considerably help on that matter.

## Supporting information

S1 File Questionnaire. (PDF)

S2 File Sample selection method. (DOCX)

S3 File The determinants of knowledge. A probit regression to assess the determinants of knowing versus not knowing at all about a disease x.
(DOCX)

S4 File Robustness checks using another definition of positive behavior. A second definition of behavior where bed net use is considered as active and not passive.
(DOCX)

S1 Text Data management and analysis. (DOCX)

S2 Text Input from LISREL. Input used to run the structural equation model in LISREL.
(DOCX)

S3 Text Structural equation model (SEM). This section details the several advantages of using SEM.
(DOCX)

S4 Text Measurement error and testing of the model. (DOCX)

S1 Fig Multiple correspondence analysis per region. (DOCX)

S1 Table Keywords cited per disease. The table exhibits all the cited keywords per disease.
(DOCX)

S2 Table Vector control measures used per disease. The table exhibits all the measures used per disease.
(DOCX)
