# Correction: Understanding the role of disease knowledge and risk perception in shaping preventive behavior for selected vector-borne diseases in Guyana

**DOI:** 10.1371/journal.pntd.0013586

**Published:** 2025-10-06

**Authors:** Céline Aerts, Mélanie Revilla, Laetitia Duval, Krijn Paaijmans, Javin Chandrabose, Horace Cox, Elisa Sicuri

S2 File, S3 File, S4 File, S1 Text and S4 Text were uploaded incorrectly. Please see the correct S2 File, S3 File, S4 File, S1 Text and S4 Text here.

## Supporting information

S1 Text (DOCX)

S4 Text (DOCX)

S2 File (DOCX)

S3 File (DOCX)

S4 File (DOCX)

## References

[1] Aerts C, Revilla M, Duval L, Paaijmans K, Chandrabose J, Cox H, et al. Understanding the role of disease knowledge and risk perception in shaping preventive behavior for selected vector-borne diseases in Guyana. PLoS Negl Trop Dis. 2020;14(4):e0008149. doi: 10.1371/journal.pntd.0008149 32251455 PMC7170267

