# Supplementary material for: Understanding the role of disease knowledge and risk perception in shaping preventive behavior for selected vector-borne diseases in Guyana
Source: PLoS Negl Trop Dis. 2020 Apr 6;14(4):e0008149. doi: 10.1371/journal.pntd.0008149 (PMC7170267; doi:10.1371/journal.pntd.0008149)
Supplement: S1 File — (PDF) [file pntd.0008149.s001.pdf]

Vector Control Services - Repuls Project

1. GPS coordinates

latitude (x.y °)

____

longitude (x.y °)

____

altitude (m)

____

précision (m)

____

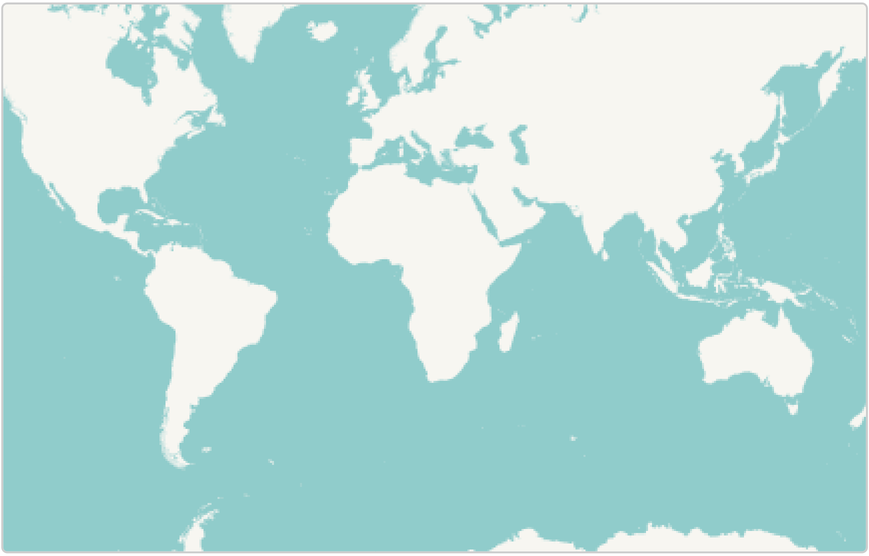

2. a. Region

- ◯ Region 1
- ◯ Region 4
- ◯ Region 6
- ◯ Region 8

2. b. Questionnaire serial number

____

3. Interviewee ID

Enter a 6-digit number after 'abc'

abc ____________________

4. Date of interview

Today's date

yyyy-mm-dd ____________________

5. Place of interview

- ◯ Private house
- ◯ Workplace
- ◯ Restaurant
- ◯ Hospital/Health centre
- ◯ School

6. Position of interviewee within the place in Question 5

- ◯ Husband
- ◯ Wife
- ◯ Workplace owner
- ◯ Workplace worker
- ◯ Hospital director
- ◯ Hospital worker
- ◯ School director
- ◯ School teacher

7. Nationality

- ◯ Guyanese
- ◯ Brazilian
- ◯ Venezuelan
- ◯ Cuban
- ◯ Other

7. b. Other nationality

____

8. Town / Village name

____

9. Sex

- ◯ Male
- ◯ Female

10. Date of birth

yyyy-mm-dd

11. What ethnic group do you belong to?

- ◯ Amerindian
- ◯ European
- ◯ African
- ◯ East Indian
- ◯ Portuguese
- ◯ Chinese
- ◯ Mixed

12. What is your martial status?

- ◯ Common law
- ◯ Married
- ◯ Separated/Divorced
- ◯ Widow/Widower
- ◯ Single

13. What is your highest level of education?

- ◯ Never been to school
- ◯ Primary
- ◯ Secondary
- ◯ Undergraduate studies
- ◯ Postgraduate studies

14. What is your occupation?

- ◯ Farmer
- ◯ Miner
- ◯ Fisherman
- ◯ Office employer
- ◯ Shop trade
- ◯ Other

14. b. Other occupation

____

15. What is your main source of drinking water?

- ◯ Piped water - Piped into dwellinger
- ◯ Piped water - Piped to yard/plot
- ◯ Piped water - Public tap/standpipe
- ◯ Piped water - Tube well or borehole
- ◯ Rainwater
- ◯ Tanker truck
- ◯ Cart with small tank
- ◯ Surface water (river/dam/lake/pond/stream/canal/irrigation channel)
- ◯ Bottled water
- ◯ Dug well
- ◯ Protected well
- ◯ Unprotected well
- ◯ Water from spring - Protected spring
- ◯ Water from spring - Unprotected spring

16. What kind of toilet facility do you use?

- ◯ No facility/bush/field
- ◯ Flush to piped sewer system
- ◯ Flush to septic tank
- ◯ Flush to pit (latrine)
- ◯ Flush to somewhere else
- ◯ Flush, don't know where
- ◯ Ventinlated improved pit latrine
- ◯ Pit latrine with slab
- ◯ Open pit
- ◯ Composting toilet
- ◯ Bucket toilet
- ◯ Hanging toilet / hanging latrine

17. Do you have:

- [ ] Electricity
- [ ] A radio
- [ ] A cell phone
- [ ] A land-line phone
- [ ] A refrigerator
- [ ] A clock
- [ ] A black/white television
- [ ] A color television
- [ ] A freezer
- [ ] An electric generator
- [ ] A fan
- [ ] An air-conditioner
- [ ] A fan
- [ ] An air-conditioner
- [ ] Washing machine
- [ ] Computer
- [ ] Digital photo-camera
- [ ] Non-digital photo-camera
- [ ] A VHS player
- [ ] A DVD player
- [ ] A bed
- [ ] A vanity
- [ ] A wall divider
- [ ] A watch
- [ ] A bicycle
- [ ] A motorbicycle
- [ ] A motorbicycle or motor scooter
- [ ] An animal-drawn cart
- [ ] A car, truck or mini-van
- [ ] A boat with a motor
- [ ] A boat without a motor

18. What type of fuel do you mainly use for cooking?

- [ ] Electricity
- [ ] LGP
- [ ] Natural gas
- [ ] Biogas
- [ ] Kerosene
- [ ] Coal, Lignite
- [ ] Charcoal
- [ ] Wood
- [ ] Straw/Shrubs/Grass
- [ ] Agricultrual crop
- [ ] Animal dung
- [ ] No food cooked in household

19. Does any member of this household own any agricultural land?

- ( ) Yes
- ( ) No

20. Which of the following animals does this household own?

- [ ] Milk cows or bulls
- [ ] Horses, donkeys or mules
- [ ] Goats
- [ ] Sheep
- [ ] Chicken or other poultry
- [ ] None of the above

20. a. How many cows?

____

20. b. How many horses, donkeys or mules?

____

20. c. How many goats?

____

20. d. How many sheep?

____

20. e. How many chickens or other poultry?

____

21. Main material of floor

- ◯ Natural - Earth/Sand
- ◯ Natural - Dung
- ◯ Rudimentary - Wood planks
- ◯ Rudimentary - Palm/bamboo
- ◯ Finished - Parquet or polished wood
- ◯ Finished - Vinyl or asphalt strips
- ◯ Finished - Ceramic tiles
- ◯ Finished - Cement
- ◯ Finished - Carpet
- ◯ Other

21. b. Other material of floor

____

22. Main material of roof

- ◯ No roof
- ◯ Natural - Thatch/palm leaf
- ◯ Natural - Sod
- ◯ Rudimentary - Rustic mat
- ◯ Rudimentary - Palm/bamboo
- ◯ Rudimentary - Wood planks
- ◯ Rudimentary - Cardboard
- ◯ Finished - Metal (including zinc)
- ◯ Finished - Wood
- ◯ Finished - Calamine/cement fiber
- ◯ Finished - Ceramic tiles
- ◯ Finished - Cement
- ◯ Finished - Roofing shingles
- ◯ Other

22. b. Other material of roof

____

23. Main material of exterior walls

- No walls
- Natural - No walls
- Natural - Cane/palm/trunks
- Natural - Dirt
- Rudimentary - Bamboo with mud
- Rudimentary - Stone with mud
- Rudimentary - Uncovered adobe
- Rudimentary - Plywood
- Rudimentary - Cardboard
- Rudimentary - Reused wood
- Finished walls - Cement
- Finished walls - Stone with lime/cement
- Finished walls - Bricks
- Finished walls - Cement blocks
- Finished walls - Covere adobe
- Finished walls - Wood planks/shingles
- Other

23. b. Other material of exterior walls

24. Does any member of your household live outside of Guyana?

- Yes
- No

24. b. Where?

24. c. What is your relation to the person(s) living outside Guyana?

- [ ] Spouse
- [ ] Son/Daughter
- [ ] Brother/Sister
- [ ] Mother/Father
- [ ] Uncle/Aunt
- [ ] Cousin
- [ ] Friend
- [ ] Other

24. d. Other relation

____

25. How many workers in total work in this place (including temporaray workers)?

____

26. What are the main activities of the place?

- [ ] Sugar
- [ ] Gold
- [ ] Bauxite
- [ ] Agriculture
- [ ] Restaurant
- [ ] Shop
- [ ] Other

26. b. Other main activity

____

27. Type of school

- ( ) Kindergarten
- ( ) Primary
- ( ) Secondary
- ( ) University
- ( ) Other

28. How many students are there in this institution?

____

29. Type of health facility

- ◯ Dispensary/Health Post
- ◯ Health Centre
- ◯ Hospital
- ◯ Private clinic

30. How many outpatients visits do you have on an ordinary day in total?

____

31. How many beds are there in the hospital?

____

32. Which is the disease you fear the most?

____

33. Do you know what the Zika virus is?

- ◯ Yes
- ◯ No

33. a. Can you briefly describe what you know about the Zika virus?

- ☐ Mosquito(es)
- ☐ Fever
- ☐ Skin rash
- ☐ Pregnancy
- ☐ Microcephaly
- ☐ Paralisis

34. Do you know what Dengue fever is?

- ◯ Yes
- ◯ No

34. b. Can you briefly describe what you know about Dengue fever?

- ☐ Mosquito(es)
- ☐ Fever
- ☐ Skin rash

35. Do you know what Malaria is?

- ◯ Yes
- ◯ No

35. b. Can you briefly describe what you know about Malaria?

- ☐ Mosquito(es)
- ☐ Fever
- ☐ Headache
- ☐ Cold sweat
- ☐ Vivax
- ☐ Falciparum

36. Do you know what Bush Yaws (Leishmaniasis) is?

- ○ Yes
- ○ No

36. a. Can you briefly describe what you know about Bush Yaws (Leishmaniasis)?

- ☐ Sandfly
- ☐ Skin lesion
- ☐ Dog

37. How much do you think you and the people in this place are at risk of Zika virus on a scale from 0 to 10 (0 - risk; 10 - very high risk)?

____

38. How much do you think you and the people in this place are at risk of Dengue on a scale from 0 to 10 (0 - risk; 10 - very high risk)?

____

39. How much do you think you and the people in this place are at risk of Malaria on a scale from 0 to 10 (0 - risk; 10 - very high risk)?

____

40. How much do you think you and the people in this place are at risk of Bush Yaws (Leishmaniasis) on a scale from 0 to 10 (0 - risk; 10 - very high risk)?

____

**41. In 5 years, what impact do you think the Zika virus will have on the health of the people of this community?**	Decrease	Remain the same	Increase	Don't know
**Zika virus**	○	○	○	○
**42. In 5 years, what impact do you think the Dengue fever will have on the health of the people of this community?**	Decrease	Remain the same	Increase	Don't know
**Dengue fever**	○	○	○	○
**43. In 5 years, what impact do you think Malaria will have on the health of the people of this community?**	Decrease	Remain the same	Increase	Don't know

	Decrease	Remain the same	Increase	Don't know
Malaria	◯	◯	◯	◯

44. In 5 years, what impact do you think Bush Yaws (Leishmaniasis) will have on the health of the people of this community?

	Decrease	Remain the same	Increase	Don't know
Bush Yaws (Leishmaniasis)	◯	◯	◯	◯

45. What would you do to avoid the Zika virus? (Multiple replies allowed)

- ☐ Nothing
- ☐ Screened windows
- ☐ Skin repellent
- ☐ Mosquito zapper racket
- ☐ Beeper mosquito
- ☐ Fogging
- ☐ Indoor residual spray
- ☐ Mosquito coils
- ☐ Bed nets
- ☐ Bracelets
- ☐ Sitting next to a fire

45. What would you do to avoid the Dengue fever? (Multiple replies allowed)

- ☐ Nothing
- ☐ Screened windows
- ☐ Skin repellent
- ☐ Mosquito zapper racket
- ☐ Beeper mosquito
- ☐ Fogging
- ☐ Indoor residual spray
- ☐ Mosquito coils
- ☐ Bed nets
- ☐ Bracelets
- ☐ Sitting next to a fire

45. What would you do to avoid Malaria? (Multiple replies allowed)

- [ ] Nothing
- [ ] Screened windows
- [ ] Skin repellent
- [ ] Mosquito zapper racket
- [ ] Beeper mosquito
- [ ] Fogging
- [ ] Indoor residual spray
- [ ] Mosquito coils
- [ ] Bed nets
- [ ] Bracelets
- [ ] Sitting next to a fire

45. What would you do to avoid Bush Yaws (Leishmaniasis? (Multiple replies allowed)

- [ ] Nothing
- [ ] Screened windows
- [ ] Skin repellent
- [ ] Mosquito zapper racket
- [ ] Beeper mosquito
- [ ] Fogging
- [ ] Indoor residual spray
- [ ] Mosquito coils
- [ ] Bed nets
- [ ] Bracelets
- [ ] Sitting next to a fire

46. How effective do you perceive the preventative measures you would use on a scale from 0 to 10?

____

SHOW INTERVIEWEE EMB1 PICTURE

Click here to upload file. (< 5MB)

SHOWINTERVIEWEE EMB2 PICTURE

Click here to upload file. (< 5MB)

SHOW INTERVIEWEE EMB3 PICTURE

Click here to upload file. (< 5MB)

SWITCH TO THE RANDOM NUMBER GENERATOR APP TO GET A NUMBER FROM 1 TO 13

Enter the number you would have generated

- ◯ 1
- ◯ 2
- ◯ 3
- ◯ 4
- ◯ 5
- ◯ 6
- ◯ 7
- ◯ 8
- ◯ 9
- ◯ 10
- ◯ 11
- ◯ 12
- ◯ 13

SHOW THE CORRESPONDING PICTURE NUMBER TO THE NUMBER YOU GENERATED

Click here to upload file. (< 5MB)

47. a. In your opinion, which of the three options described do you think is best?

- ◯ EMB1
- ◯ EMB2
- ◯ EMB3

47. b. Taking into account your circumstances, which one of the options would you take?

- ◯ EMB1
- ◯ EMB2
- ◯ EMB3
- ◯ None
