# Supplementary material for: Understanding the role of disease knowledge and risk perception in shaping preventive behavior for selected vector-borne diseases in Guyana
Source: PLoS Negl Trop Dis. 2020 Apr 6;14(4):e0008149. doi: 10.1371/journal.pntd.0008149 (PMC7170267; doi:10.1371/journal.pntd.0008149)
Supplement: S2 File — (DOCX) [file pntd.0008149.s002.docx]

S2. Sample selection method

Sample size was strongly determined by the availability of resources: our sampling approach was therefore exploratory. Within this context, we aimed at representing both coastal and hinterland regions: the two categories of regions summarize substantial within-country variability across a number of relevant dimensions for this study. Coastal regions are more populated with a high concentration of the main country towns (Georgetown, the country capital, and New Amsterdam). In the coastal regions, there is a higher burden of *Aedes* mosquitos transmitted parasite infections (such as dengue and zika viruses) and access to health care services is much easier than in the hinterland. Hinterland regions have an unusual low population density, with nearly the totality of its inhabitants concentrated in a few small towns. The economy in the hinterland is mostly based on extraction activities (mainly of gold, diamonds and bauxite) and logging. The access to healthcare is poor and the burden of vector borne diseases is mainly determined by parasites transmission though *Anopheles* mosquitos (malaria). Given our resources constraints, we chose two coastal and two hinterland regions. Coastal regions include regions 4 and 6, the former includes the capital city Georgetown which concentrates 30% of the national population while the latter includes the second largest city, New Amsterdam. Interior regions consisted of regions 1 and 8, which are geographically vast but with a very low population density, and where we conducted our survey around two main towns: Mabaruma and Mahdia respectively.

Again, given the limited resources available, we collected about 800 questionnaires in total, which we equally divided across the 4 regions (i.e. a sample size per region of 200). We obtained information from the Bureau of Statistics of Guyana on the number of inhabitants in each region, split by lower administrative units (called villages) – most recent population data were from the 2012 national census. Based on that information, a subset of villages were selected by the Ministry of Public Health based on their proximity to the starting point and their accessibility by walking distance or public transport. More precisely, 49 villages out of 198 villages were ‘preselected’ for region 4 and 22 out of 190 villages for region 6. Among these villages, 15 villages in regions 4 and 6 were randomly chosen applying sampling proportional to size [1]. In each of the selected 15 neighborhoods, the number of questionnaires was also assigned based on population’s size, with more questionnaires assigned to more populated neighborhoods (Table A1). We assigned 210 questionnaires to account for potential attrition^[[1]](#footnote-1)^. A starting point was chosen by data collectors within each neighborhood who then applied the “spinning bottle” rule [2]. In the interior of the country, since most villages are small, far away from each other and poorly connected, we decided to focus our research around the main centers of Mabaruma in region 1 and Mahdia in region 8, which have a total population of about 2,000 inhabitants each. For those two towns, selection proportional to size was not applied. Instead, data collectors selected houses starting from the health center and then moved forward applying the “spinning bottle” rule.

| Table S2: Selection proportional to size (regions 4 and 6) | | | |
| --- | --- | --- | --- |
| Names (or abbreviations) of your primary sampling units | Estimated size of sampling units | Probability of inclusion | Number of quesitonnaires |
| **Region 4** | | | |
| Cummings Lodge | 7246 | 1 | 21 |
| Kitty | 6789 | 1 | 20 |
| Turkeyen | 6599 | 0,978557447 | 19 |
| Campbellville | 5031 | 0,74604069 | 18 |
| Pattensen | 5013 | 0,743371493 | 17 |
| West Ruimveldt | 4206 | 0,623702473 | 16 |
| Albouystown | 3838 | 0,569132214 | 15 |
| Sophia | 3687 | 0,546740613 | 14 |
| Liliandaal | 3100 | 0,459695118 | 13 |
| Werk En Rust | 2760 | 0,409276944 | 12 |
| Albertown | 2357 | 0,349516579 | 11 |
| East La Penitence | 1984 | 0,294204876 | 10 |
| Ogle | 1391 | 0,206269648 | 9 |
| Prashad Nagar | 1013 | 0,150216502 | 8 |
| Lamaha Gardens | 638 | 0,094608221 | 7 |
| **Region 6** | | | |
| Cumberland | 3875 | 1 | 21 |
| Mount Sinai | 3861 | 1 | 20 |
| Canefield | 3268 | 1 | 19 |
| Rose Hall | 3067 | 1 | 18 |
| Stanleytown | 3049 | 1 | 17 |
| Glasgow | 1868 | 0,94448377 | 16 |
| Adelphi | 1303 | 0,658812822 | 15 |
| Little Bleyendaal | 1214 | 0,613813328 | 14 |
| Sheet Anchor | 1114 | 0,563252098 | 13 |
| Edinburg | 1069 | 0,540499545 | 12 |
| Reliance | 921 | 0,465668925 | 11 |
| Ordnance Fort Canje | 883 | 0,446455658 | 10 |
| Overwinning | 873 | 0,441399535 | 9 |
| Vrymans Erven | 802 | 0,405501062 | 8 |
| Palmyra or No. 4 | 540 | 0,27303064 | 7 |
| *NB: 210 questionnaires were aimed in each region to account for dropouts in the following rounds of data collection* | | | |

1. The study is currently proceeding with 2 additional data collection rounds among the same cohort of individuals. [↑](#footnote-ref-1)
