# Supplementary material for: Understanding the role of disease knowledge and risk perception in shaping preventive behavior for selected vector-borne diseases in Guyana
Source: PLoS Negl Trop Dis. 2020 Apr 6;14(4):e0008149. doi: 10.1371/journal.pntd.0008149 (PMC7170267; doi:10.1371/journal.pntd.0008149)
Supplement: S4 File — A second definition of behavior where bed net use is considered as active and not passive. (DOCX) [file pntd.0008149.s004.docx]

S4. Robustness check by using another definition of positive behavior

In this case, behavior is equal to 0 if the respondent uses nothing, 1 if the respondent uses IRS and/or fogging, 2 if the respondent uses one other measure than IRS and/or fogging, 3 if the respondent uses two other measures than IRS and/or fogging, etc. Accordingly, as opposed to the definition used in the manuscript, bed net use is no longer considered passive but active. Results of the SEM are provided in the below Table A5.

| Table S9: Robustness check. Results of the structural model with the other definition of behavior | | | | | |
| --- | --- | --- | --- | --- | --- |
|  |  | **Malaria** | **Dengue fever** | **Cutaneous leishmaniasis** | **Zika virus** |
| **Dependent variable** | **Explanatory variables** | St. Coeff  (Std. Error) | St. Coeff  (Std. Error) | St. Coeff  (Std. Error) | St. Coeff  (Std. Error) |
| **Equation 1** | | | | | |
| **Behavior** | Knowledge | 0.924***  (0.096) | 0.382***  (0.116) | 0.999***  (0.301) | -0.627  (0.403) |
|  | Risk | 0.267*  (0.135) | = | = | = |
|  | Wealth | 0.155***  (0.055) | -0.217***  (0.073) | = | = |
|  | Region | -0.563***  (0.109) | = | -0.219  (0.199) | 0.178  (0.252) |
|  | Educ | 0.038  (0.047) | = | -0.127*  (0.073) | = |
|  | Female | 0.041  (0.035) | = | = | = |
| **Equation 2** | | | | | |
| Knowledge | Wealth | 0.017  (0.045) | = | -0.226**  (0.085) | 0.208**  (0.075) |
|  | Region | 0.630***  (0.059) | 0.359***  (0.068) | -0.523***  (0.104) | 0.569***  (0.078) |
|  | Educ | 0.168***  (0.036) | = | = | = |
|  | Female | 0.011  (0.032) | = | = | = |
| **Equation 3** | | | | | |
| Risk | Knowledge | -0.110  (0.120) | 0.094  (6.873) | -0.114  (0.769) | = |
|  | Behavior | -0.071  (0.138) | = | 1.481**  (0.607) | = |
|  | Wealth | 0.030  (0.049) | -0.027  (0.144) | = | = |
|  | Region | 0.924***  (0.078) | 0.366  (2.470) | = | 0.019  (0.130) |
|  | Educ | 0.088  (0.058) | -0.077  (1.155) | = | 0.035  (0.095) |
|  | Female | -0.007  (0.043) | = | = | = |
| N |  | 438 | 335 | 134 | 231 |
| Chi-Squared (df)=34.70 (23); p-value=0.05566 | | | | | |
| RMSEA=0.042 | | | | | |
| Legend: ‘=’ implies that coefficients are equal to the ones estimated for the malaria model (model 1); **significant at 5% significance level; *** significant at 1% significance level; St. Coeff= standardized coefficient; Std. Error= Standard error; N=sample size; df= degrees of freedom; RMSEA= Root Mean Square Error of Approximation. | | | | | |
