# Supplementary material for: Understanding the role of disease knowledge and risk perception in shaping preventive behavior for selected vector-borne diseases in Guyana
Source: PLoS Negl Trop Dis. 2020 Apr 6;14(4):e0008149. doi: 10.1371/journal.pntd.0008149 (PMC7170267; doi:10.1371/journal.pntd.0008149)
Supplement: S1 Text — (DOCX) [file pntd.0008149.s005.docx]

**S1 Text. Data management and analysis**

The data collected were uploaded either instantaneously or at the end of the day (depending on the availability of the network facilities) in an online reporting platform (<https://www.kobotoolbox.org/>) that could only be accessed with a password. All questionnaires were coded with a unique serial number and each interviewee was identified with a unique code to ensure anonymity of the data. Data were primarily exported into Microsoft Excel and afterward exported to Stata software (StataCorpLP, <http://www.stata.com>) to obtain the correlation matrices, which were then inputted into LISREL (<http://www.ssicentral.com/lisrel/>) for estimating the SEM using the below input.
