# Supplementary material for: Understanding the role of disease knowledge and risk perception in shaping preventive behavior for selected vector-borne diseases in Guyana
Source: PLoS Negl Trop Dis. 2020 Apr 6;14(4):e0008149. doi: 10.1371/journal.pntd.0008149 (PMC7170267; doi:10.1371/journal.pntd.0008149)
Supplement: S2 Text — Input used to run the structural equation model in LISREL. (DOCX) [file pntd.0008149.s006.docx]

S2 Text. Input from LISREL

Group1 Malaria

data ng=4 ni=7 no=438 ma=km

km

1.0000

0.3508 1.0000

0.0194 0.2140 1.0000

0.2073 0.0227 -0.1048 1.0000

0.1900 -0.0093 -0.0083 0.2797 1.0000

0.0455 0.4351 0.6193 -0.2331 -0.0350 1.0000

0.0386 -0.0834 -0.0195 0.0605 0.0995 -0.0365 1.0000

labels

Behavior Know Risk Educ Wealth Region Female

se

Behavior Know Risk Wealth Region Educ Female /

model ny=3 nx=4 ne=3 nk=4 lx=fu,fi ly=fu,fi te=fu,fi td=fu,fi be=fu,fi ga=fu,fi ph=sy,fr ps=sy,fi

le

Behavior Know Risk

lk

Wealth Region Educ Female

!measurement error

va 0.728 ly 1 1

va 0.747 ly 2 2

va 0.752 ly 3 3

!fix variance of errors terms to 1-q2

va .47 te 1 1

va .4420 te 2 2

va .4345 te 3 3

!effects between the eta

fr be 1 2 be 3 2 be 1 3 be 3 1

!effects between eta and ksi

fr ga 1 1 ga 1 2 ga 2 2 ga 3 2 ga 2 3 ga 1 3 ga 3 3 ga 2 1 ga 1 4 ga 2 4 ga 3 4 ga 3 1

!measurement perfect for the x

va 1 lx 1 1

va 1 lx 2 2

va 1 lx 3 3

va 1 lx 4 4

!free variances for the eta

fr ps 1 1 ps 2 2 ps 3 3

out mi AD=OFF it=500

Group2 Dengue

data ni=7 no=335 ma=km

km

1.0000

0.2047 1.0000

-0.0239 0.0888 1.0000

0.1397 0.1663 -0.0972 1.0000

-0.0248 0.0819 -0.0382 0.3201 1.0000

-0.2223 0.2440 0.3305 -0.1692 -0.0500 1.0000

0.0233 0.0211 -0.0239 0.0616 0.1396 -0.0674 1.0000

labels

Behavior Know Risk Educ Wealth Region Female

se

Behavior Know Risk Wealth Region Educ Female /

model ny=3 nx=4 ne=3 nk=4 lx=fu,fi ly=fu,fi te=fu,fi td=fu,fi be=in ga=in ph=sy,fr ps=in

va 0.728 ly 1 1

va 0.747 ly 2 2

va 0.752 ly 3 3

va .47 te 1 1

va .4420 te 2 2

va .4345 te 3 3

va 1 lx 1 1

va 1 lx 2 2

va 1 lx 3 3

va 1 lx 4 4

fr ga 3 2 ga 3 1 ga 2 2 ga 1 1

fr ps 2 1 ps 3 1 ps 3 3

out mi AD=OFF it=500

Group3 leishmaniasis

data ni=7 no=134 ma=km

km

1.0000

0.4425 1.0000

0.1499 0.3525 1.0000

0.0890 0.0265 0.1042 1.0000

-0.1540 -0.1572 -0.0027 0.3275 1.0000

-0.5240 -0.4257 -0.1796 -0.0830 0.1078 1.0000

0.0384 0.0732 0.1299 0.1081 0.0947 -0.0759 1.0000

labels

Behavior Know Risk Educ Wealth Region Female

se

Behavior Know Risk Wealth Region Educ Female /

model ny=3 nx=4 ne=3 nk=4 lx=fu,fi ly=fu,fi te=fu,fi td=fu,fi be=in ga=in ph=sy,fr ps=in

va 0.728 ly 1 1

va 0.747 ly 2 2

va 0.752 ly 3 3

va .47 te 1 1

va .4420 te 2 2

va .4345 te 3 3

va 1 lx 1 1

va 1 lx 2 2

va 1 lx 3 3

va 1 lx 4 4

fr be 1 2 be 3 1 be 3 2

fr ga 2 1 ga 2 2 ga 1 2 ga 2 3

fr ps 2 1 ps 3 1 ps 2 2 ps 3 2

out mi AD=OFF it=500

Group4 zika

data ni=7 no=231 ma=km

km

1.0000

0.0928 1.0000

0.0290 -0.0633 1.0000

0.0271 0.2371 0.0329 1.0000

0.0370 0.2188 0.0171 0.1344 1.0000

-0.0047 0.4272 -0.0238 -0.0655 0.1270 1.0000

-0.0121 0.0728 -0.0442 -0.0489 0.0713 -0.0993 1.0000

labels

Behavior Know Risk Educ Wealth Region Female

se

Behavior Know Risk Wealth Region Educ Female /

model ny=3 nx=4 ne=3 nk=4 lx=fu,fi ly=fu,fi te=fu,fi td=fu,fi be=in ga=in ph=sy,fr ps=in

va 0.728 ly 1 1

va 0.747 ly 2 2

va 0.752 ly 3 3

va .47 te 1 1

va .4420 te 2 2

va .4345 te 3 3

va 1 lx 1 1

va 1 lx 2 2

va 1 lx 3 3

va 1 lx 4 4

fr be 1 2

fr ga 2 1 ga 1 2 ga 2 2 ga 3 2 ga 3 3 ga 3 1

fr ps 1 1 ps 2 2 ps 3 2 ps 3 3

pd

out mi AD=OFF it=500
