# Supplementary material for: Understanding the role of disease knowledge and risk perception in shaping preventive behavior for selected vector-borne diseases in Guyana
Source: PLoS Negl Trop Dis. 2020 Apr 6;14(4):e0008149. doi: 10.1371/journal.pntd.0008149 (PMC7170267; doi:10.1371/journal.pntd.0008149)
Supplement: S3 Text — This section details the several advantages of using SEM. (DOCX) [file pntd.0008149.s007.docx]

### S3 Text. Structural equation model (SEM)

SEM has been widely used in social sciences, initially among quantitative scientists in sociology and psychology [3] and later became one of the causal models for health-sciences research [4]. Despite the causality debate surrounding the use of SEM [5], this model has often been suggested to deal with the different sources of endogeneity without requiring longitudinal data. First, it is capable of dealing with omitted variable bias (as long as these do not play a crucial role in the analysis) by allowing correlation between the error terms [3]. An example of omitted variable in here is whether the individual experienced a previous episode of the disease, which is likely to be correlated with the error term of *knowledge*, *risk* perception and *behavior*. Second, SEM is able to account for measurement error by using latent variables as indicators of observed variables. Indeed, measurement error can be significant when using reported measures and particularly when related to health [6]. Third, this model may be capable of solving for reverse relationship, which is conditional on being empirically identified (i.e. from having at least as many exogenous variables than endogenous ones). Fourth, SEM allows for comparing models in terms of their best fit with the data – the so-called ‘confirmatory analysis’ [7]. Lastly, another feature of SEM is that it allows for multiple group analysis so that statistical differences between groups (i.e. diseases) can be assessed [8]. Hence, we are able to test whether the responsiveness of preventive behavior to risk differs across diseases.
