# Supplementary figures and images for: Understanding the role of disease knowledge and risk perception in shaping preventive behavior for selected vector-borne diseases in Guyana
Source: PLoS Negl Trop Dis. 2020 Apr 6;14(4):e0008149. doi: 10.1371/journal.pntd.0008149 (PMC7170267; doi:10.1371/journal.pntd.0008149)

S1 Figure: Multiple correspondence analysis per region

**
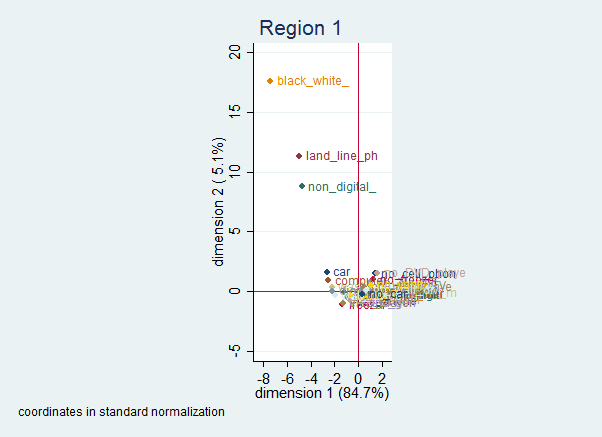
**

**
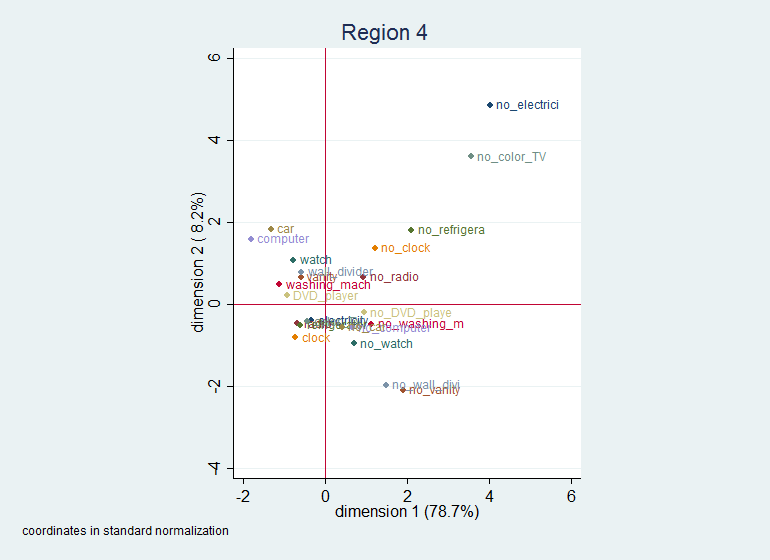
**

**
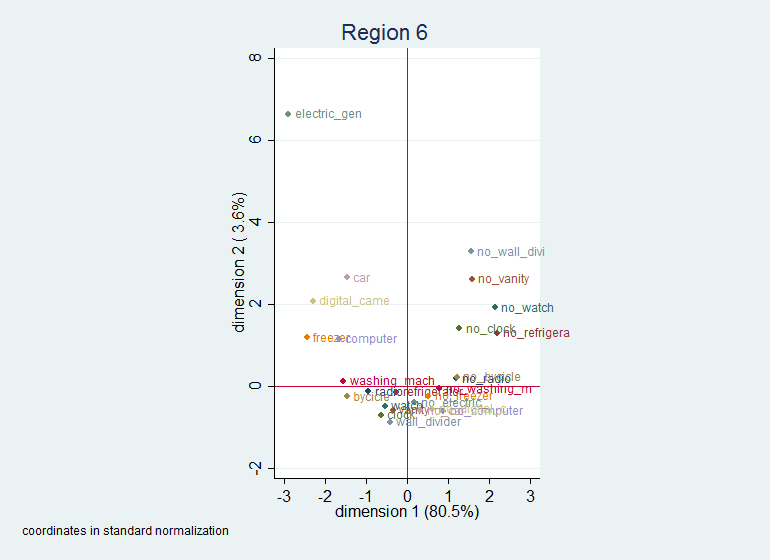
**


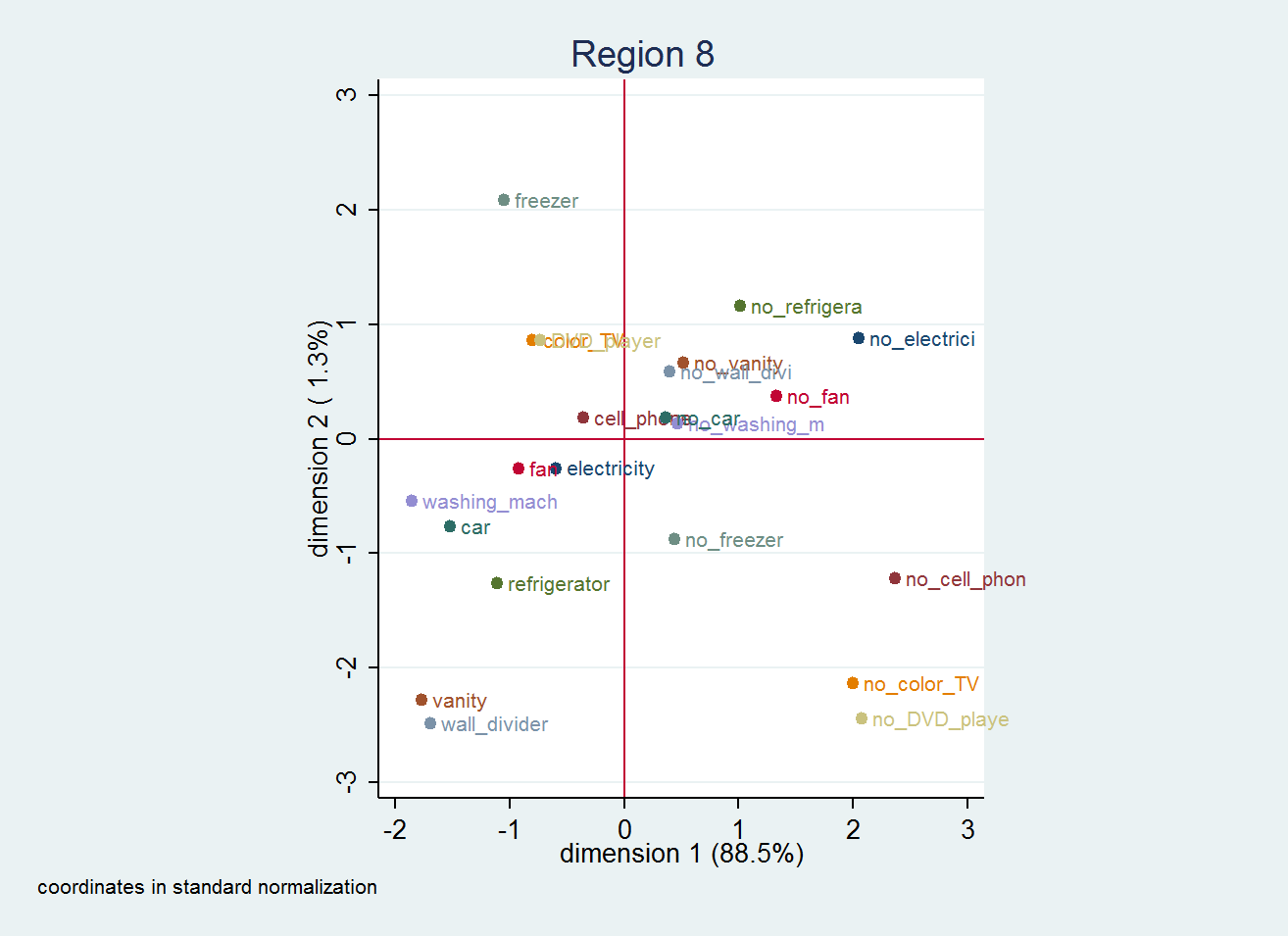

Supplement: S1 Fig — (DOCX) [file pntd.0008149.s009.docx]
