# Supplementary material for: Understanding the role of disease knowledge and risk perception in shaping preventive behavior for selected vector-borne diseases in Guyana
Source: PLoS Negl Trop Dis. 2020 Apr 6;14(4):e0008149. doi: 10.1371/journal.pntd.0008149 (PMC7170267; doi:10.1371/journal.pntd.0008149)
Supplement: S1 Table — The table exhibits all the cited keywords per disease. (DOCX) [file pntd.0008149.s010.docx]

| S1 Table: Keywords cited per disease | |
| --- | --- |
| **Malaria** | |
| *Do you know malaria? If yes, could you please describe what you know about malaria?* | |
| **Variables** | **% (N=497)** |
| Do not know | 11.87% |
| **keywords** |  |
| Mosquito(es) | 25.68% |
| Fever | 24.09% |
| Headache | 18.73% |
| Cold sweat | 13.44% |
| Vivax | 8.99% |
| Falciparum | 9.06% |
| **Dengue fever** | |
| *Do you know dengue fever? If yes, could you please describe what you know about dengue fever?* | |
| **Variables** | **% (N=497)** |
| Do not know | 32.6% |
| **keywords** |  |
| Mosquito(es) | 49.32% |
| Fever | 47.16% |
| Skin rash | 3.52% (18) |
| **Zika virus** | |
| *Do you know zika virus? If yes, could you please describe what you know about zika virus?* | |
| **Variables** | **% (N=497)** |
| Do not know | 53.52% |
| **keywords** |  |
| Mosquito(es) | 50.83% |
| Fever | 18.05% |
| Pregnancy | 13.30% |
| Microcephaly | 13.06% |
| Skin rash | 3.80% |
| Paralysis | 0.95% |
| **Cutaneous leishmaniasis (CL)** | |
| *Do you know cutaneous leishmaniasis? If yes, could you please describe what you know about cutaneous leishmaniasis?* | |
| **Variables** | **% (N=497)** |
| Do not know | 77.46% |
| **keywords** |  |
| Skin lesion | 82.69% |
| Sandfly | 16.67% |
| Dog | 0.64% |
| Legend: freq.=frequency | |
