# Supplementary material for: Understanding the role of disease knowledge and risk perception in shaping preventive behavior for selected vector-borne diseases in Guyana
Source: PLoS Negl Trop Dis. 2020 Apr 6;14(4):e0008149. doi: 10.1371/journal.pntd.0008149 (PMC7170267; doi:10.1371/journal.pntd.0008149)
Supplement: S2 Table — The table exhibits all the measures used per disease. (DOCX) [file pntd.0008149.s011.docx]

| S2 Table: Vector control measures used per disease  *What do you do to avoid disease x?* | | | | | |
| --- | --- | --- | --- | --- | --- |
|  |  | **Malaria** | **Dengue fever** | **Zika virus** | **Cutaneous leishmaniasis** |
| Provided by the government | Bed nets | 87.67% | 68.36% | 63.20% | 23.88% |
|  | Indoor residual spray | 27.40% | 35.22% | 37.23% | 20.90% |
|  | Fogging | 13.47% | 17.31% | 9.52% | 14.18% |
| Not provided by the government | Skin repellent | 26.48% | 21.49% | 39.83% | 11.94% |
|  | Mosquito coils | 34.93% | 15.82% | 19.48% | 3.73% |
|  | Screened windows | 4.34% | 2.39% | 4.33% | 1.49% |
|  | Mosquito zapper racket | 2.28% | 2.09% | 3.46% | 1.49% |
|  | Sitting next to a fire | 0.68% | 0.30% | 0.43% | 0 |
|  | Bracelets | 0.68% | 0 | 0 | 0 |
|  | Beeper mosquito | 0.23% | 0 | 0.43% | 0 |
|  | Legend: freq.= frequency | | | | |
