# Supplementary material for: Correction: Understanding the role of disease knowledge and risk perception in shaping preventive behavior for selected vector-borne diseases in Guyana
Source: PLoS Negl Trop Dis. 2025 Oct 6;19(10):e0013586. doi: 10.1371/journal.pntd.0013586 (PMC12500141; doi:10.1371/journal.pntd.0013586)
Supplement: S4 Text — (DOCX) [file pntd.0013586.s002.docx]

**S4 Text. Measurement error and testing of the model**

Since measurement error can be significant for survey measures, it was considered for the self-reported variables: *behavior*, *knowledge* and *risk* perception using the Survey Quality Predictor program (<http://sqp.upf.edu/>) [9]–[11]. Furthermore, since SEM is better at dealing with continuous variables rather than categorical ones, correcting for measurement error also takes into account that while the latent variable is continuous the observed variable behind may be categorical. For the exogenous variables – *wealth*, *education*, *region*, and *female* – it is not possible to correct for measurement error using the SQP program and hence it was assumed a perfect measurement, implying a quality coefficient of 1. Nonetheless, this is not believed to be a major issue as these variables do not embed subjectivity.

Regarding the testing of the model, parameters of the equations were first assumed equal across diseases (i.e. group analysis) in LISREL. Afterward, based on Jrule suggestions – a postestimation tool for the SEM command that indicates local misspecifications based on the modification index (MI), the power of the MI and the expected parameter change (EPC) [12]–[14] – parameters that were found to differ across diseases were let free to vary. Jrule was used in addition to the usual chi-square test to assess the model’s fit because – as opposed to the latter – it is able to detect the size of the misspecification and is not influenced by sample size [15].
