# Supplementary material for: Correction: Understanding the role of disease knowledge and risk perception in shaping preventive behavior for selected vector-borne diseases in Guyana
Source: PLoS Negl Trop Dis. 2025 Oct 6;19(10):e0013586. doi: 10.1371/journal.pntd.0013586 (PMC12500141; doi:10.1371/journal.pntd.0013586)
Supplement: S3 File — (DOCX) [file pntd.0013586.s004.docx]

**S3. The determinants of knowledge:**

Knowledge_malaria_i_ = β_0_ + β_1_Education_i_ + β_2_Sex_i_ + β_3_Wealth_index_i_ + β_4_ Region_i_ + ε_i_

Knowledge_dengue_i_ = β_0_ + β_1_Education_i_ + β_2_Sex_i_ + β_3_Wealth_index_i_ + β_4_ Region_i_ + ε_i_

Knowledge_zika_i_ = β_0_ + β_1_Education_i_ + β_2_Sex_i_ + β_3_Wealth_index_i_ + β_4_ Region_i_ + ε_i_

Knowledge_leishmaniasis_i_ = β_0_ + β_1_Education_i_ + β_2_Sex_i_ + β_3_Wealth_index_i_ + β_4_ Region_i_ + ε_i_

Where i=individual 1 to 497

| Table S3: The determinants of knowledge (probit regression) | | | | |
| --- | --- | --- | --- | --- |
|  | Malaria | Dengue fever | Zika virus | Cutaneous leishmaniasis |
|  | Coef.  (St. Err) | Coef.  (St. Err) | Coef.  (St. Err) | Coef.  (St. Err) |
| **Education** |  |  |  |  |
| No formal education (base) | (empty) |  |  |  |
|  |  |  |  |  |
| Primary education | -1.329** | -0.382 | 0.918 | 0.196 |
|  | (0.540) | (0.710) | (0.587) | (0.469) |
| Secondary education | -0.836 | -0.106 | 1.449** | 0.373 |
|  | (0.518) | (0.710) | (0.588) | (0.471) |
| University | (base) | -0.324 | 1.617** | 0.349 |
|  |  | (0.768) | (0.635) | (0.542) |
| **Sex** |  |  |  |  |
| Female (base) |  |  |  |  |
|  |  |  |  |  |
| Male | 0.558** | 0.407** | 0.0320 | 0.0812 |
|  | (0.247) | (0.169) | (0.147) | (0.154) |
| **Wealth index** |  |  |  |  |
| 1^st^ quantile of the wealth index (base) |  |  |  |  |
|  |  |  |  |  |
| 2^nd^ quantile of the wealth index | 0.340 | -0.285 | 0.342* | 0.282 |
|  | (0.288) | (0.219) | (0.198) | (0.226) |
| 3rd quantile of the wealth index | 0.260 | -0.123 | 0.640*** | 0.627*** |
|  | (0.269) | (0.211) | (0.201) | (0.226) |
| 4rth quantile of the wealth index | 0.107 | -0.180 | 0.666*** | 0.882*** |
|  | (0.281) | (0.217) | (0.204) | (0.223) |
| 5th quantile of the wealth index | 0.370 | -0.0828 | 0.706*** | 0.649*** |
|  | (0.306) | (0.223) | (0.203) | (0.223) |
| **Region** |  |  |  |  |
| region 4 (base) |  |  |  |  |
|  |  |  |  |  |
| region 6 | -0.630*** | -0.751*** | -0.303* | -0.470** |
|  | (0.194) | (0.173) | (0.171) | (0.226) |
| region 8 | 0.988*** | -0.0800 | -0.764*** | 0.557*** |
|  | (0.280) | (0.171) | (0.179) | (0.185) |
| region 1 | (empty) | 1.586*** | 0.584*** | 0.962*** |
|  |  | (0.252) | (0.168) | (0.177) |
| Constant | 1.639*** | 0.620 | -1.794*** | -1.850*** |
|  | (0.584) | (0.713) | (0.589) | (0.472) |
| Observations | 347 | 497 | 497 | 497 |

Standard errors in parentheses

*** p<0.01, ** p<0.05, * p<0.1

The coefficient cells are empty for the categories *No education* and *region 1* in the malaria estimation reducing the sample size to 347 individuals. This is because there were no people with no education who had no knowledge of malaria and there were no people living in region 1 who did not know about malaria. As “no formal education” is empty in the malaria equation, “University” becomes the reference category for education.
